# Supplementary material for: Spectral Modulation of Optofluidic Coupled-Microdisk Lasers in Aqueous Media
Source: Nanomaterials (Basel). 2019 Oct 11;9(10):1439. doi: 10.3390/nano9101439 (PMC6835252; doi:10.3390/nano9101439)
Supplement: Supplementary file 1 [file nanomaterials-09-01439-s001.pdf]

*nanomaterials*

*Article*

# Spectral Modulation of Optofluidic Coupled-Microdisk Lasers in Aqueous Media

**Zhihe Guo [1], Haotian Wang [2,*], Chenming Zhao [1], Lin Chen [1], Sheng Liu [1], Jinliang Hu [1], Yi Zhou [1] and Xiang Wu [1,*]**

[1] Key Laboratory of Micro and Nano Photonic Structures (Ministry of Education), Department of Optical Science and Engineering, Shanghai Engineering Research Center of Ultra Precision Optical Manufacturing, Fudan University, Shanghai 200433, China; 17110720004@fudan.edu.cn (Z.G.); 13110720009@fudan.edu.cn (C.Z.); 13110720001@fudan.edu.cn (L.C.); 14110720003@fudan.edu.cn (S.L.); 14110720001@fudan.edu.cn (J.H.); 18110720008@fudan.edu.cn (Y.Z.)

[2] Jiangsu Key Laboratory of Advanced Laser Materials and Devices, School of Physics and Electronic Engineering, Jiangsu Normal University, Xuzhou 221116, China

* Correspondence: wanghaotian@jsnu.edu.cn (H.W.); wuxiang@fudan.edu.cn (X.W.)

Received: 2 September 2019; Accepted: 9 October 2019; Published: date

**Supplementary Materials:**

**I. Fabrication of the solid-state dye-doped polymer microdisks**

**II. Fabrication of the optofluidic microdisk device**

**III. Relationship between the refractive index of DMSO-water mixtures and the DMSO (volume fraction)**

**IV. Relationship between the refractive index *n* and wavelength shift**

## I. Fabrication of the solid-state dye-doped polymer microdisks

The solid-state dye-doped polymer microdisk arrays were fabricated via single-mask standard lithography, as shown in Figure S1. The geometric shapes of the single and coupled microdisks were pre-designed on the chromium mask, and the gap between each pair of coupled microdisks was increased from 0.2 to 1 μm, and 0.1 μm for each pair. The diameter of the microdisk was about 20 μm and the thickness was 2.4 μm, as measured using scanning electron microscopy (SEM) and surface profiler, respectively.

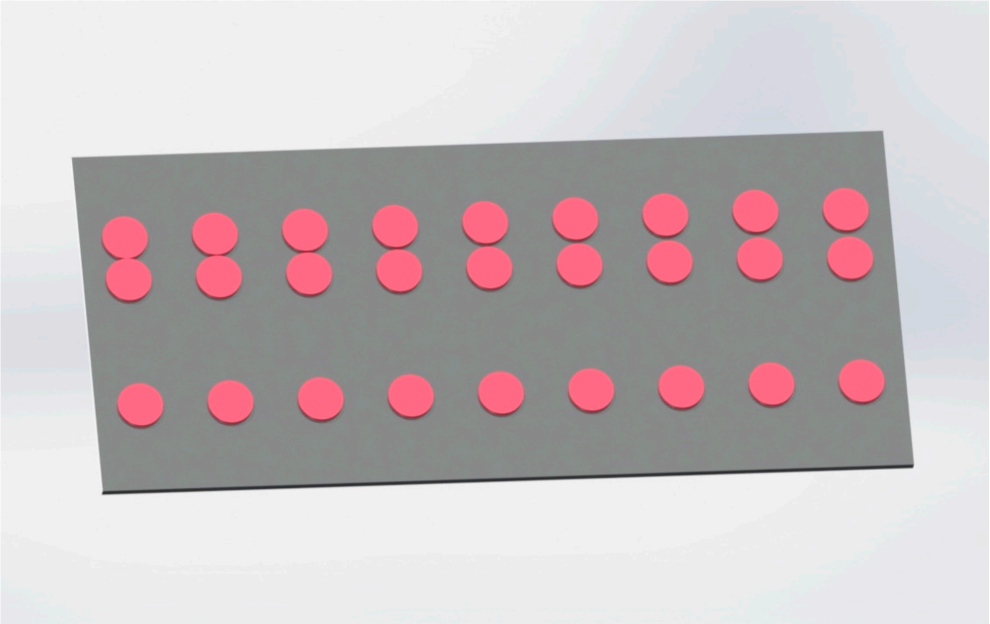

**Figure 1.** 3-D schematic of the solid-state dye-doped polymer microdisks.

## II. Fabrication of the optofluidic microdisk device

Figure S2 plotted the fabrication process of the optofluidic microdisk device. This microfluidic channel was a device combines a clean glass slide and patterned polydimethylsiloxane (PDMS) leaf. The patterned PDMS leaf was formed from a glass mold and released after 2 h of 95 °C curing in an oven. The shape of the microfluidic channel was shown in in Figure S2c. The optofluidic microdisk device, consisting of a microfluidic channel and solid-state dye-doped polymer microdisks, was fabricated and as shown in Figures 2d. Finally, an optofluidic microdisk device, consisting of a microfluidic channel and solid-state dye-doped polymer microdisks, was fabricated and as shown in Figure S2e.

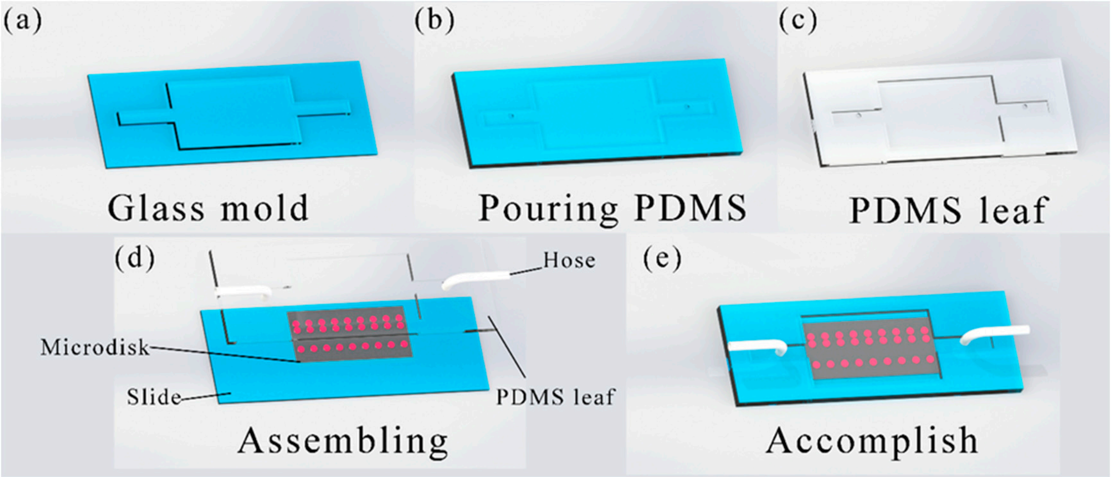


**Figure 2.** 3-D schematic diagram shows the fabrication process of the optofluidic microdisk device.

## III. Relationship between the refractive index of DMSO-water mixtures and the DMSO (volume fraction)

Actually, the refractive indices of the solutions in the experiment are calculated by the volume ratio of DMSO to deionized water [36]. The relationship between the DMSO-water mixtures and the refractive indices is due to Lorentz-Lorenz and can be described as:

$$\frac{n_{12}^2-1}{n_{12}^2+2}=\phi_1\frac{n_1^2-1}{n_1^2+2}+\phi_2\frac{n_2^2-1}{n_2^2+2}, \quad (0)$$

where, $n_{12}$, $n_1$ and $n_2$ are the refractive indices of the DMSO-water mixtures, DMSO and DI-water, respectively. $\phi_1$ and $\phi_2$ are the volume fractions of the DMSO and DI-water in the mixture. Using Equation S1, the refractive index of DMSO-water mixtures can be calculated.

**IV. Relationship between the refractive index *n* and wavelength shift**

The relationship between resonator wavelength ($\lambda$) and the $n_{eff}$ is $m\lambda = 2\pi n_{eff}R$ [37], where $m$ is angular momentum, $n_{eff}$ is the effective refractive index of the WGMs inside the resonator, R is the radius of the resonator. When the refractive index of background medium is changing, it will cause the $n_{eff}$ changes, leading to a WGM wavelength shift.

© 2019 by the authors. Submitted for possible open access publication under the terms and conditions of the Creative Commons Attribution (CC BY) license (http://creativecommons.org/licenses/by/4.0/).
